# Teleosts as Model Organisms To Understand Host-Microbe Interactions

**DOI:** 10.1128/JB.00868-16

**Published:** 2017-07-11

**Authors:** Emily A. Lescak, Kathryn C. Milligan-Myhre

**Affiliations:** University of Alaska Anchorage, Department of Biological Sciences, Anchorage, Alaska, USA; Geisel School of Medicine at Dartmouth

**Keywords:** gnotobiotic, animal models, fish, host-microbe interactions, microbiota

## Abstract

Host-microbe interactions are influenced by complex host genetics and environment. Studies across animal taxa have aided our understanding of how intestinal microbiota influence vertebrate development, disease, and physiology. However, traditional mammalian studies can be limited by the use of isogenic strains, husbandry constraints that result in small sample sizes and limited statistical power, reliance on indirect characterization of gut microbial communities from fecal samples, and concerns of whether observations in artificial conditions are actually reflective of what occurs in the wild. Fish models are able to overcome many of these limitations. The extensive variation in the physiology, ecology, and natural history of fish enriches studies of the evolution and ecology of host-microbe interactions. They share physiological and immunological features common among vertebrates, including humans, and harbor complex gut microbiota, which allows identification of the mechanisms driving microbial community assembly. Their accelerated life cycles and large clutch sizes and the ease of sampling both internal and external microbial communities make them particularly well suited for robust statistical studies of microbial diversity. Gnotobiotic techniques, genetic manipulation of the microbiota and host, and transparent juveniles enable novel insights into mechanisms underlying development of the digestive tract and disease states. Many diseases involve a complex combination of genes which are difficult to manipulate in homogeneous model organisms. By taking advantage of the natural genetic variation found in wild fish populations, as well as of the availability of powerful genetic tools, future studies should be able to identify conserved genes and pathways that contribute to human genetic diseases characterized by dysbiosis.

## INTRODUCTION

In vertebrates, the gut microbiome promotes the normal development of host physiology (1), skeletal systems (2, 3), and metabolism (4) while decreasing susceptibility to pathogens. It is sensitive to disruptions (5) that are often associated with short- and long-term consequences for host health and development (2, 6), such as inflammatory bowel disease, type II diabetes, colorectal cancer, autoimmune diseases, and autism (7, 8). Vertebrates harbor complex residential microbial communities that have been shaped by the host (9) and, unlike invertebrate models such as fruit flies and squid, have adaptive immune systems that recognize particular microbes and play vital roles in cultivating residential gut microbial communities (9). While studies of host-microbe interactions have provided novel insights into development, disease, and physiology, gaps remain in our understanding of the processes underlying microbial community assembly (10, 11), the mechanisms by which gut microbes influence host development and physiology (12–18), and the genetic and environmental factors that regulate gut microbial composition and diversity (19). Bridging these gaps requires the development of robust, versatile, and genetically tractable model systems (20).

## COMPARISON OF MOUSE AND FISH MODELS

Inbred mouse models have traditionally been used to study host-microbe interactions. More than 450 strains have been described since the first inbred mice were created nearly 100 years ago. These strains are valuable not only because of their isogenicity, which allows the isolation of a particular genetic variant of interest, but also because phenotypic differences among strains have been described in great detail (21). The rich collection of knockout, knock-in, and mutant lines has greatly increased understanding of how host genetics contribute to microbial community composition, immune function, and metabolism (22).

However, studies using mouse models have been restricted in several ways. Use of inbred lines limits understanding of how complex genetic variation influences microbial community composition (23). For example, at least 163 genetic loci of small effect in the human genome have been linked to irritable bowel disorder (24), and many of them serve purposes with respect to immune system signaling and mucosal barrier integrity across vertebrates (25, 26). Disrupting these genes individually and/or in various combinations would require a staggering number of mouse lines. Genetic differences have often accumulated between mutant and wild-type colonies that had been separately maintained for multiple generations, leading to discordant results among strains reared at different laboratories (27). When genetically variable individuals are used, husbandry constraints can result in small sample sizes and limited statistical power (19).

In addition to genetic constraints, the inability to observe microbe interactions in live mice can prevent in-depth studies of host-microbe interactions. Most mouse studies rely upon indirect characterization of gut microbial communities from fecal samples, which are not consistently reliable indicators of gut microbial communities (28–34) and cannot be used to detect differences in microbial communities that are spatially separated along the gut (32). Additional concerns include whether observations made under artificial conditions are actually reflective of what occurs in the wild (20, 35). These limitations highlight the need for model systems that allow robust statistical examination of how microbial communities are shaped by complex natural host genetic variation (36) in both laboratory-reared and wild populations.

The 28,000 characterized fish species comprise nearly half of all vertebrate diversity and possess extensive variation in physiology, ecology, and natural history (37) that can facilitate our understanding of the evolution of host-microbe interactions (38). Relative to the contribution of fish species to overall vertebrate diversity, their microbial communities have remained underexplored (39), although they have been characterized in a range of fishes (see, e.g., references 40, 41, 42, and 43).

Teleosts possess physiological and immunological features common to all vertebrates as well as a complex gut microbiota. Both teleosts and mammals have a digestive tract consisting of a liver, gallbladder, pancreas, and intestine that develop in a similar trajectory, from the rostral gut to the hindgut and midgut. Guts are separated along the rostral-caudal axis and have an intestinal epithelium made up of absorptive enterocytes, secretory goblet cells, and enteroendocrine cells (44). Intestines initially form in a sterile environment and complete their development in the presence of microbes (14). In much the same way that mammalian newborns are first colonized by microbes at birth, fish initially acquire their gut microbes from the environment upon opening of the digestive tract, which typically occurs a couple of days after hatching (45). Gut microbes aid in fermentation of polysaccharides to short-chain fatty acids (46) and protect against pathogenic infection (47, 48). The genes involved in immune system signaling are highly conserved between mammals and teleosts, as well (26, 49, 50).

Teleost physiology and mammalian physiology also differ in several ways. Teleosts lack lymph nodes and bone marrow (51), although the head kidney is considered orthologous in function. The teleost innate immune system is more diverse than that of mammals, but their immunoglobulins have fewer antibodies (52–54). While a great diversity of gut microbes has been sampled across fish species, most communities have been dominated by the Proteobacteria (20, 38, 55–57). This is in contrast to healthy mammalian guts, which are dominated by Bacteroidetes and Firmicutes (22). An exception has been documented in herbivorous marine fishes, which closely resemble herbivorous mammalian guts, suggesting that their microbial communities share similar functions in gut fermentation (38).

Aside from physiological and microbial community differences between fishes and mammals, fish models also present some experimental constraints. The roles of early life exposures that have both short- and long-term consequences on gut microbial community structure in mammals, such as mode of delivery (vaginal versus cesarean) and breast milk (58), cannot be studied in teleosts. Humanized microbiome mice models (59) allow the transplantation of human microbes into mice to recapitulate some aspect of their host's phenotype and are a valuable tool for understanding the influence of the gut microbiome in disease and the role of diet in shaping the microbiome (60, 61). This technique has not been developed in fish.

## ADVANTAGES OF ZEBRAFISH AND THREESPINE STICKLEBACK MODELS

**FIG 1:**
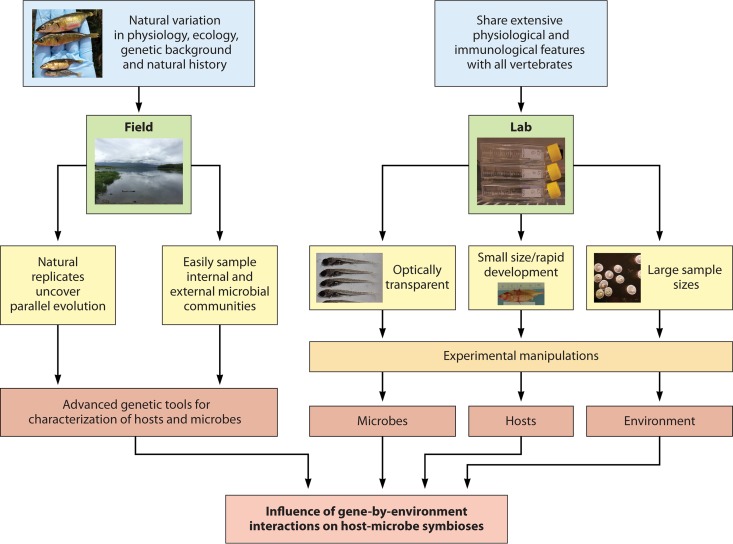
Teleosts exhibit natural variation, and their physiology is remarkably similar to that of other vertebrates, including humans. These features have led to a rich history of study that has made teleosts strong model organisms for both field and laboratory studies of host-microbe interactions. The ease with which their internal and environmental microbial communities can be sampled, their adaptive radiations, and the availability of advanced genetic tools for characterizing hosts and microbes have made them ideal for empirical studies. In the laboratory, the optical transparency, large sample sizes, small size, and rapid development of fish have facilitated experimental manipulations of microbes and hosts and their environment. Combining field and laboratory studies allows identification of gene-environment interactions influencing host-microbe symbioses.

Most of the host-microbe research using teleosts has focused on zebrafish (Danio rerio). However, threespine stickleback (Gasterosteus aculeatus), which is a widely used model organism in evolution, genetics, and ecology, has recently also been adapted for host-microbe interaction research. Advantages of these two systems lie in the powerful genetic tools that have been developed and their rich history of study, dating back to the 1800s (62) (Fig. 1). Single crosses produce a large number of offspring that can be housed in highly controlled environments (63) and permit statistically robust studies; their rapid development and small size have made them valuable resources for a wide range of genetic studies; and both their internal and external (environmental) microbial communities can be easily sampled and manipulated (20, 50, 56), unlike those of mammals. A powerful asset of these models lies in the ability to study the evolution of the relationship between the host and its microbiota due to the host's relatively short life span (1 to 2 years) and the extensive knowledge that we have of laboratory lines (zebrafish) and wild populations (stickleback). Coupled with annotated genomes and the ability to compare host and microbial DNA and transcriptomes, these teleosts have already begun to advance our understanding of host-microbe interactions.

The transparency of zebrafish eggs and juveniles allowed the first successful examination of the colonization dynamics of bacteria within live, developing hosts (14). The ability to genetically manipulate both host cells and microbes to express fluorescent proteins allows real-time nondestructive observations of spatial and temporal variation in host-microbe interactions in developing zebrafish, which has granted insights into the distribution of bacterial populations along the gut (12), complex microbial behaviors (64), and population dynamics during colonization (65). However, this technique is limited to genetically modifiable microbes, which represent only a fraction of the community present in fish guts.

**FIG 2:**
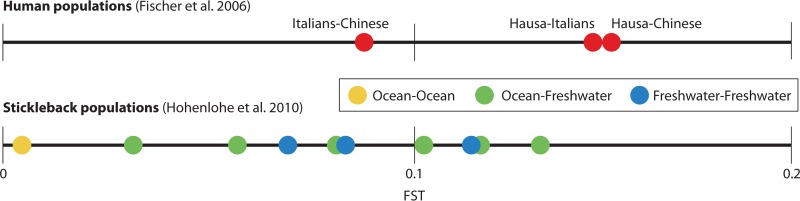
The threespine stickleback is an appropriate model organism for studying the effects of host genetic background on microbial community because wild populations exhibit genetic variation that is comparable to that of human populations. *F*_ST_, a measure of genetic divergence, among human populations ranges from about 0.08 to 0.15 (132) (top panel) and among stickleback populations from Alaska ranges from almost 0 to 0.13 (133) (bottom panel).

A primary advantage of using threespine stickleback as a model organism is the ability to study how natural genetic variation, which is of a magnitude similar to that found in the human population (Fig. 2), influences a range of phenotypes, including bone development (66), pigmentation (67, 68), and behavior (69). Many genetic regions, such as those associated with skeletal structures, also underlie variations in human populations (70). Stickleback therefore present a great potential to reveal genes important for driving microbial membership and the host response to microbes, including processes involved in metabolic changes, cell development, and cell-to-cell signaling.

## GNOTOBIOTIC STUDIES IN TELEOSTS

In gnotobiotic studies, animals are derived under germfree conditions and analyzed either in this sterile state or in association with specific microbes in comparison to conventionally reared animals with diverse microbial communities (71). Gnotobiotic techniques are straightforward in fishes since they develop *ex utero* and eggs can be surface sterilized shortly after fertilization (13). Gnotobiotic techniques were first developed in platyfish (Xiphophorus maculatus), followed by tilapia (Tilapia macrocephala), salmon (Salmo salar), sheepshead minnow (Cyprinidon vairegatus), Atlantic halibut (Hippoglossus hippoglossus), and turbot (Scopthalmus maximus) (72–77). However, the most detailed studies of host-microbe interactions have used gnotobiotic zebrafish (78, 79) and stickleback (80). Gnotobiotic studies in both mammals and teleosts allow the documentation of a broad array of host responses to gut microbiota (13), but zebrafish have revealed advantages over mice in identifying microbial signaling pathways influencing development (14, 56). However, while multigenerational gnotobiotic lines are able to be maintained in mice (22), this is not yet possible in fishes.

Gnotobiotic studies in zebrafish have revealed that the gut microbiota stimulates intestinal epithelial cell proliferation (13, 14) through MyD88 signaling pathways (15) and promotes shifts in epithelial glycan expression (14) as well as recruitment of gut-associated immune cells (13, 16, 81). Germfree zebrafish intestines have decreased secretory cell numbers and experience faster peristaltic contractions than conventionally reared individuals (14). Their guts are unable to fully develop and exhibit reduced function, but these deficiencies can be reversed after introduction of bacteria (14, 16). These studies reveal the varied roles that microbiota play in normal digestive development and function.

Gnotobiotic studies of laboratory-reared oceanic and resident freshwater stickleback have demonstrated that these two ecotypes have common gut microbial communities and similarities in intestinal development, despite their separation in the wild for at least 10,000 years (80). However, the two ecotypes differed in the intensity of their inflammatory responses to microbes, highlighting the potential for gene-environment interactions that influence host immune response (80).

## INSIGHTS FROM WILD-CAUGHT VERSUS LABORATORY-REARED ZEBRAFISH

Wild-caught and laboratory-reared zebrafish populations have similar gut microbial communities, suggesting the existence of a core gut microbiota (20), which may also be true of mammals (82–84). However, neutral processes of drift and dispersal can generate a great deal of diversity within and among individuals. Bacterial taxa that deviate from neutral patterns and are more widespread than expected are likely adapted to, and selected by, the host (63). These examples highlight the utility of genetically variable model organisms that can be studied both in the wild and under controlled laboratory conditions to examine how gene-environment interactions drive microbial community dynamics.

## INSIGHTS FROM WILD-STICKLEBACK POPULATIONS

The colonization of thousands of lakes throughout the Northern Hemisphere by oceanic ancestral stickleback resulted in an adaptive radiation of freshwater populations that are locally adapted to their environments. This “natural experiment” allows researchers to study the influences of environmental factors, such as water chemistry and predation regimes, on the evolution of a vertebrate host (134, 135). Host-microbe researchers are now beginning to use the natural variation found in wild populations to unravel interactions among diet, genetic background, and environmental microbial communities with respect to effects on gut microbiota composition. Such studies have revealed that microbial community structure appears to be more strongly driven by differences in host genotype than by differences in environment (85) and that food-associated microbes drive gut microbial community diversity to a greater extent than water-associated microbes (86). Inverse relationships between diversity in major histocompatibility complex class II (MHC-II) alleles and diversity in gut microbial community suggest that adaptive immunity could restrict the diversity of commensal bacteria. Sex also influences the degree and direction of influence of the MHC-II receptors as well as the magnitude of effects of diet on microbiota composition (100): males have higher phylogenetic diversity than females, and phylogenetic diversity increases with size more strongly in males than in females. While associations have also been found between MHC diversity and microbiota in mice (87), microbiota changes correlated more strongly with body size in females than in males (88). Sex differences across taxa are likely due to interactions among hormones, developmental rate, and/or gene expression, which are all mechanisms that can be readily examined in laboratory experiments.

## INSIGHTS FROM OTHER FISH SPECIES

The changes in community composition that occur during development and migration in salmonids present the opportunity to explore how gene-environment interactions shape the microbiome (89). A study of wild Atlantic salmon (Salmo salar) revealed differences between environmental and gut microbial communities that were driven largely by ontogeny rather than geography (90). The intestinal microbiota of rainbow trout (Oncorhynchus mykiss) has been shown to be highly variable temporally, spatially, and interindividually (55, 91). Seasonal fluctuations in temperature were correlated with changes in gut microbiota (92) in both rainbow trout and gulf killifish (Fundulus grandis), with decreased bacterial counts in winter and increases in spring that were associated with rising temperature (93, 94). Seasonal differences have also been documented in wild-mouse populations (95).

Studies have also explored how antimicrobials change fish gut bacterial community composition (96). For example, low levels of triclosan exposure resulted in differences in microbial community structure in the fathead minnow (Pimephales promelas) (97). However, the communities recovered to baseline after 2 weeks in clean water, suggesting that short-term disruption to gut microbiota may be sufficient to harm a developing host but that there is an opportunity to recover normal bacterial diversity after disturbance.

## CONCLUSIONS AND FUTURE DIRECTIONS

### What have we learned from studying fish models?

Researchers have gained novel insights into mechanisms underlying development of the digestive tract and how microbiota contribute to disease states (13–18, 80, 81, 86, 90) (Table 1). We have learned from studies of fishes and other vertebrates that gut microbiota are dynamic and demonstrate complex successional patterns throughout development (58, 101–103). Differences in microbial communities between captive fishes and their wild counterparts argue for the use of model systems, such as threespine stickleback, that can be studied in the wild as well as under controlled laboratory conditions (98). Perhaps surprisingly, fish gut communities more closely resemble those of mammals than those of organisms found in their environment (38, 104), particularly with regard to abundances of Proteobacteria, Firmicutes, and Bacteroidetes (38, 99, 105, 106), which further promotes the idea of their utility as model organisms for human health research.

**TABLE 1:** Studies using teleosts as model organisms have made major contributions to understanding host-microbe interactions

Contribution	Reference(s)
Contributions of microbiota to host development	
Stimulation of intestinal epithelial cell proliferation through MyD88 signaling pathways	13–15
Promotion of a shift in epithelial glycan expression	14
Stimulation of recruitment of immune cells	13, 16
Promotion of gut development	14
Maintenance of normal levels of secretory cells and peristaltic contractions	14, 16
Aiding in host growth and development	15, 17, 18
Process of gut colonization	
Bacterial populations not uniformly distributed along gut	12
Establishment of bacteria during development	13, 14, 56
Quantification of bacterial population dynamics in a living host	65
Gene-environment interactions	
Core gut microbiota	20, 80
Taxa that deviate from neutral patterns are more likely adapted to, and selected by, host environment	63
Microbiota more strongly driven by differences in host genotype than environment	85
Diet and host genetics influence on microbiota	18, 86, 98
Microbiota influenced more by host developmental stage than geography	90
Sex influences magnitude of relationship to diet	86
Temporal, spatial, and interindividual variation	20, 55, 91, 99
Seasonal variation in microbiota	92, 93
Immune system-microbiota interactions	
Variation in strength of inflammatory response to microbes in genetically divergent populations	80
Correlations between MHC class II alleles and microbiota	100
Microbiota-induced neutrophil recruitment	81
Effects of antimicrobials: low levels of triclosan alter microbial community structure	97

### Where do we go from here?

Teleost systems can be used to identify selective pressures, including interactions among environment, diet, genetic background, and development, that influence gut microbial community assembly. For example, interactions among MHC diversity, sex, and diet raise the issue of how hormones, sexual dimorphisms, metabolism, and gene expression influence host-microbe interactions and susceptibility to disease. Epistatic interactions among a large number of genes can be difficult to characterize or manipulate in an inbred model, highlighting the utility of model organisms, such as threespine stickleback, that exhibit complex natural genetic variation. Taking advantage of the natural genetic variation found in wild fish populations as well as the availability of powerful genetic tools, future studies should be able to identify conserved genes and pathways that contribute to human genetic diseases characterized by dysbiosis (107–129).

Studies examining the effects of exposure to antibiotics and other contaminants suggest that juvenile dysbiosis can impact long-term fitness in contaminated habitats (97). While previous work has focused on how clinical levels of antibiotics (130) and antimicrobials (97) affect the abundance of specific taxa, what remains largely unknown is how environmentally relevant levels of common contaminants may disrupt the microbiota, resulting in developmental abnormalities and/or disease. Stickleback are already common model organisms for understanding the effects of chronic exposure to aquatic contaminants on physiological development (136) and can therefore easily be used to understand the effects of exposure to environmentally relevant levels of aquatic pollutants on gut microbial community and host development. The conservation of physiological and genetic pathways among vertebrates will allow insights into the environmental factors that may trigger dysbiosis in humans, as well.

### How many teleost models do we need?

Since fishes exhibit dramatic variations in physiology, natural history, and ecology, they can be used as model organisms to address a wide range of factors relevant to host-microbe interactions. For example, studies of fishes that are of economic and cultural significance, such as salmonids, have potential to improve aquaculture (131) and safe harvesting practices and to contribute to our understanding of how populations may respond to climate and anthropogenic changes. Focusing on widespread species that have undergone adaptive radiations, such as whitefish (Coregonus), will allow further insight into the relative influences of phylogeny and environment in shaping microbial communities. Fishes living in extreme environments, such as Death Valley pupfish (Cyprinodon salinus) and Antarctic icefish (Notothenioidei), can help us understand how microbes may enable vertebrates to adapt to extreme environments. Finally, live-bearing fishes have advantages in understanding colonization dynamics early in development. Now that so much is known about how microbial communities influence many aspects of a host's life, including its physiology, immune response, and behavior, fish models can help us better understand the effects of microbial community diversity and disruption on host development and adaptation to its environment.
